# Antiflammatory activity and potential dermatological applications of characterized humic acids from a lignite and a green compost

**DOI:** 10.1038/s41598-022-06251-2

**Published:** 2022-02-09

**Authors:** Mariavittoria Verrillo, Melania Parisi, Davide Savy, Giuseppina Caiazzo, Roberta Di Caprio, Maria Antonietta Luciano, Sara Cacciapuoti, Gabriella Fabbrocini, Alessandro Piccolo

**Affiliations:** 1 Centro Interdipartimentale di Ricerca per la Risonanza Magnetica Nucleare per l’Ambiente, l’Agroalimentare, ed i Nuovi Materiali (CERMANU), Università di Napoli Federico II, Via Università 100, 80055 Portici, Italy; 2 Department of Clinical Medicine and Surgery, Università di Napoli Federico II, Naples, Italy

**Keywords:** Environmental sciences, Medical research, Green chemistry, Supramolecular chemistry

## Abstract

Long-term exposure to air pollution has been associated with the development of some inflammatory processes related to skin. The goal of modern medicine is the development of new products with antiflammatory action deriving from natural sources to improve environmental and economic sustainability. In this study, two different humic acids (HA) were isolated from from lignite (HA-LIG) and composted artichoke wastes (HA-CYN) and characterized by infrared spectrometry, NMR spectroscopy, thermochemolysis-GC/MS, and high-performance size-exclusion chromatography (HPSEC), while their antiflammatory activity was evaluated on HaCaT cells. Spectroscopic results showing the predominance of apolar aliphatic and aromatic components in HA-LIG, whereas HA-CYN revealed a presence of polysaccharides and polyphenolic lignin residues. The HA application on human keratinocyte pre-treated with Urban Dust revealed a general increase of viability suggesting a protective effect of humic matter due to the content of aromatic, phenolic and lignin components. Conversely, the gene expression of IL-6 and IL-1β cytokines indicated a significant decrease after application of HA-LIG, thus exhibiting a greater antiflammatory power than HA-CYN. The specific combination of HA protective hydrophobic components, viable conformational arrangements, and content of bioactive molecules, suggests an innovative applicability of humic matter in dermatology as skin protectors from environmental irritants and as antiflammatory agents.

## Introduction

In the last decades, global air quality has shown a progressive decline, a direct effect of human activities such as biomass burning, industrial operations and vehicle emissions^1^. The World Health Organization (WHO) estimated that air pollution was implicated into 3.7 million deaths representing more than 10% of all-cause deaths in industrialized countries^2^, thereby rendering air pollution as the world's largest single environmental health risk^3^.

Recently, large epidemiological studies identified that exposure to air-borne pollution increases respiratory and cardiovascular morbidity^4,5^. Moreover, long-term exposure to air pollution has been associated with the development of certain cancers and may affect some processes related to skin, including aging, and heighten the clinical manifestations of psoriasis, acne, and atopic dermatitis^6^. In fact, skin is considered the first barrier of human body to the external factors and is constantly exposed to harmful compounds existing in the surrounding environment^7^.

The most common air pollutants comprise polycyclic aromatic hydrocarbons (PAHs), volatile organic compounds (VOCs, e.g., benzene), particulate matter (PM, most commonly PM2.5 and PM10), gaseous pollutants, (carbon-monoxide, nitric oxides, sulphur oxide, ozone, heavy metals) and indoor pollutants, as solid fuels consumption^8^. Although the molecular mechanisms relating air pollutions to skin diseases are not yet fully understood, several in vivo studies have highlighted the occurrence and prominence of the inflammatory state as a common factor of these conditions^9^. Particularly, the activation of a multiprotein complex called inflammasome, has been hypothesized to trigger the inflammatory responses by promoting the maturation of proinflammatory cytokines^10^. Therefore, the possible inhibition of inflammatory markers in the skin can represent an excellent defence against the toxic effects of air pollution^11^. Currently, one of the objectives of modern medicine is the development of new products with antiflammatory activities deriving from natural sources, to improve the environmental and economic sustainability of innovative effective therapies becoming complementary to traditional ones^12^.

While several natural substances such as flavonoids and polyphenol derivatives play a healing role on the damage induced by inflammation^13–15^, the use of non-steroidal antiflammatory agents may promote a side uncontrolled reaction and increase gastrointestinal toxicity risk as well as cardiovascular disease^16^. Therefore, an increasing attention is devoted to search for innovative products to reduce and mitigate the consequences of inflammatory processes. In this context, natural organic derivates, such as humic acids (HA), found several applications in different pharmaceutical fields like medicine and veterinary science due to the acknowledged antiviral, antimicrobial, profibrinolytic, antiflammatory and estrogenic activities^17,18^. Moreover, these bioactive molecules can be easily isolated from recycled biomasses such as green compost from agricultural wastes, thus widening the value of recycled biomasses within the circular economy framework^18,19^. Some humic materials like fulvic acids have been used in the topical treatment of dermatitis and psoriasis, rheumatoid arthritis and eczema^20,21^. Although, some scientific evidences have shown a relation between application of humic substances and release of cytokines through the activation of a superoxide dismutase mechanism or the migration and adhesion of cells of the damaged sites, the inherent structure–activity relationship have not been clearly elucidated^22^.

The aim of this study was to evaluate two different humic acids extracted from lignite and composted artichoke wastes as antiflammatory effectors, following the exposure of HaCaT cells to atmospheric particulate matter. The molecular properties of humic materials were analysed in detail to gain insight of the bioactive components and implement a comprehensive understanding of the molecular bases of their potential dermatological application.

## Results and discussion

### Elemental content in HA-LIG and HA-CYN

The elemental composition of humic acids revealed a significant larger C content (56%) in the geochemically derived HA-LIG, while the C/N ratio highlighted the preferential incorporation of nitrogen-containing compounds (e.g.: peptides) in HA-CYN (Table 1). The H/C values suggested a prevalent preservation of apolar components in both HA, which were preferentially aromatic and aliphatic in HA-LIG and HA-CYN, respectively (Table 1).

**Table 1:** Elemental composition (%) of HA-LIG and HA-CYN.

%	Total C	Total N	Total H	C/N ratio^a^	H/C ratio^a^
HA-LIG	56 ± 2	2 ± 0.04	4 ± 1	32.67	0.86
HA-CYN	29.1 ± 2.2	2.52 ± 0.0	3.79 ± 0.2	13.44	1.53

^a^Atomic ratio

### Infrared spectroscopy

**Figure 1:**
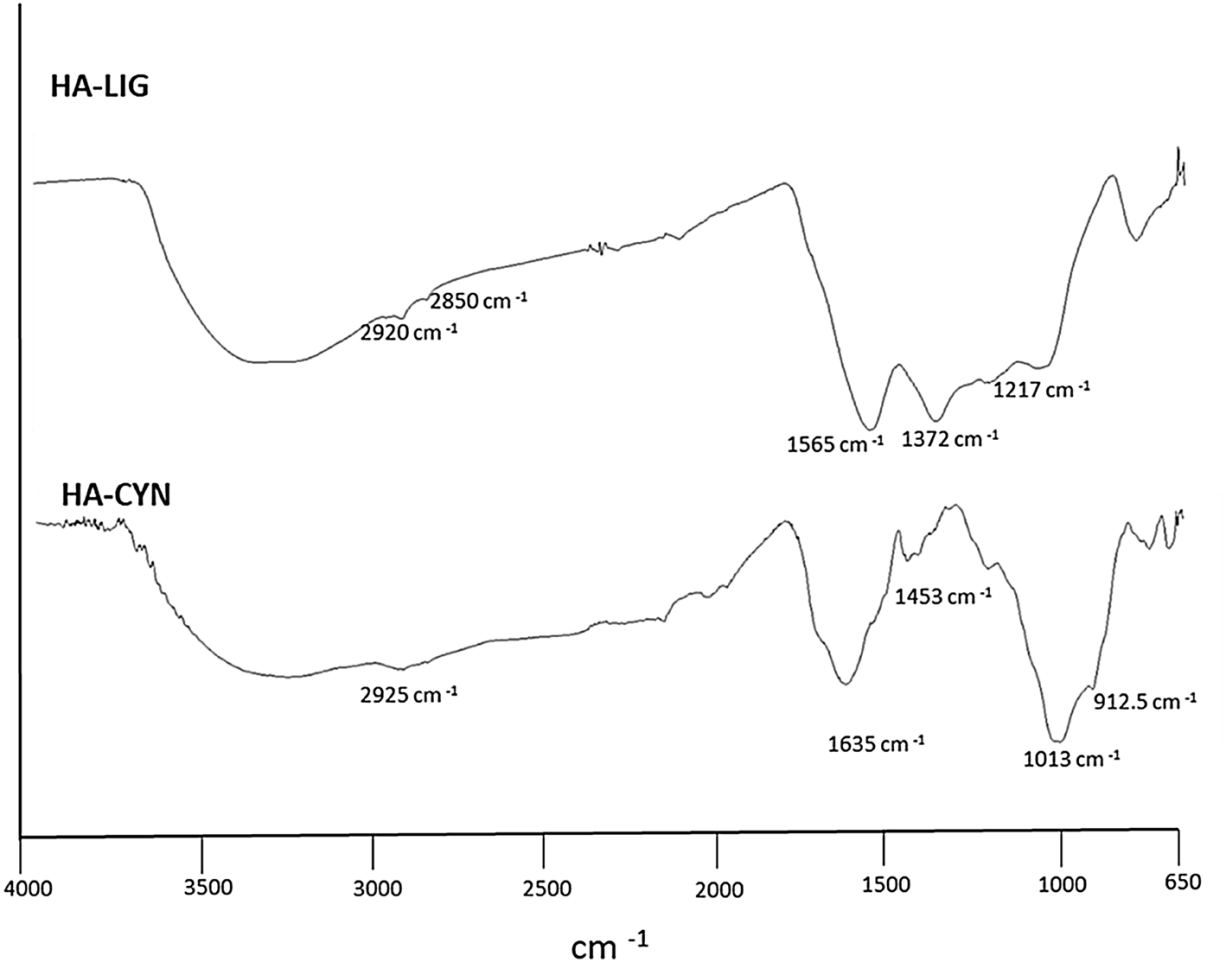
FTIR-ATR spectra of Humic acids from lignite (HA-LIG) and from green compost made of artichoke residues (HA-CYN).

The ATR spectra of HA-LIG and HA-CYN showed poorly diagnostic broad bands in the 3200–3400 cm^−1^ range, assigned to O–H and N–H stretching vibrations (Fig. 1). Both HA samples were characterized by shoulders at 2850 and 2920 cm^−1^, associated to symmetric and asymmetric stretching vibration of CH_2_ and CH_3_ groups in long chain alkyl compounds^23^. The main infrared differences between the two HA were shown at smaller wavenumbers (Fig. 1). The prominent peak at 1565 cm^−1^ in the HA-LIG spectrum is related to ring vibrations in aromatic structures, while the less intense signal ay 1372 and 1217 cm-^1^ are currently assigned to C–O bending of ethers and phenolic compounds^24^. In the HA-CYN spectrum, the sharp wide band at around 1635 cm^−1^ may include both C=O stretching vibrations of carboxylate groups of alkanoic acids and the vibrations of conjugated and unconjugated double C=C bonds^23,25^. The small signals at 1450 and 1220 cm^−1^ derive from the bending of C–H and C–O groups in alkyl chains and phenolic components, respectively. Finally, a large incorporation of carbohydrates derivatives in HA-CYN from green compost was indicated by the intense band around 1013 cm^−1^_,_ due to the C–O stretchings in the alcohol functions in pyranoside rings^25^.

### Off-line Pyr-TMAH-GC–MS

The pyrogram of HA-LIG revealed a typical profile of a humic acid of geochemical origin characterized almost exclusively by alkyl and aromatic apolar components (Figure S1)^26^. The main aromatic molecules identified in the first part of the spectrum, were related to benzene, toluene, *m*- and *p*-xylene, styrene, naphthalene and alkyl-naphthalene, phenolic structures, lignin compounds and benzoic acids. The peaks at longer retention times were assigned to long alkyl chains of alkanoic acids and alkane/alkene doublets (Table S1). The data of HA-LIG revealed its main hydrophobic features, with long alkyl chains and aromatic moieties being currently ascribed to thermal degradation products of selectively preserved apolar fractions. These components are due to the advanced structural modification of natural compounds during the accumulation processes of geo-organic sediments^27^.

The pyrogram of HA-CYN was mainly composed by ester and ether derivatives of lignin fragments and linear and branched chain fatty acids followed by biopolyesters, carbohydrates derivatives and N-containing compounds (Table S2). The most abundant lignin monomers were the oxidized products of both di- and tri-methoxy phenylpropane molecules, with aldehydic-, ketonic-, benzoic-acid, and benzene-propenoic acids as main components (Figure S1; Table S2). This finding combined with the lower yields of less altered lignin units indicated the prevalent incorporation in HA-CYN of bio-available lignin fragments from composted plant residues^18,19,25^. In the wide range of long chain alkanoic acids (Table S1), the largest abundance was that of the ubiquitous hexadecanoic and octadecanoic homologues, while a prevalence of even-carbon linear chains suggests a plant origin^25^. The presence of branched chain fatty acids found in the HA-CYN pyrogram was related to natural microbial constituents built up in the mature compost from which HA-CYN was extracted^25^. In respect to HA-LIG, the larger abundance of polar compounds in HA-CYN included methylated forms of mono- and oligosaccharides components and nitrogenated compounds, such as peptide, indole, and pyrrole derivatives (Table S2).

### ^13^C-CPMAS NMR spectra

**Figure 2:**
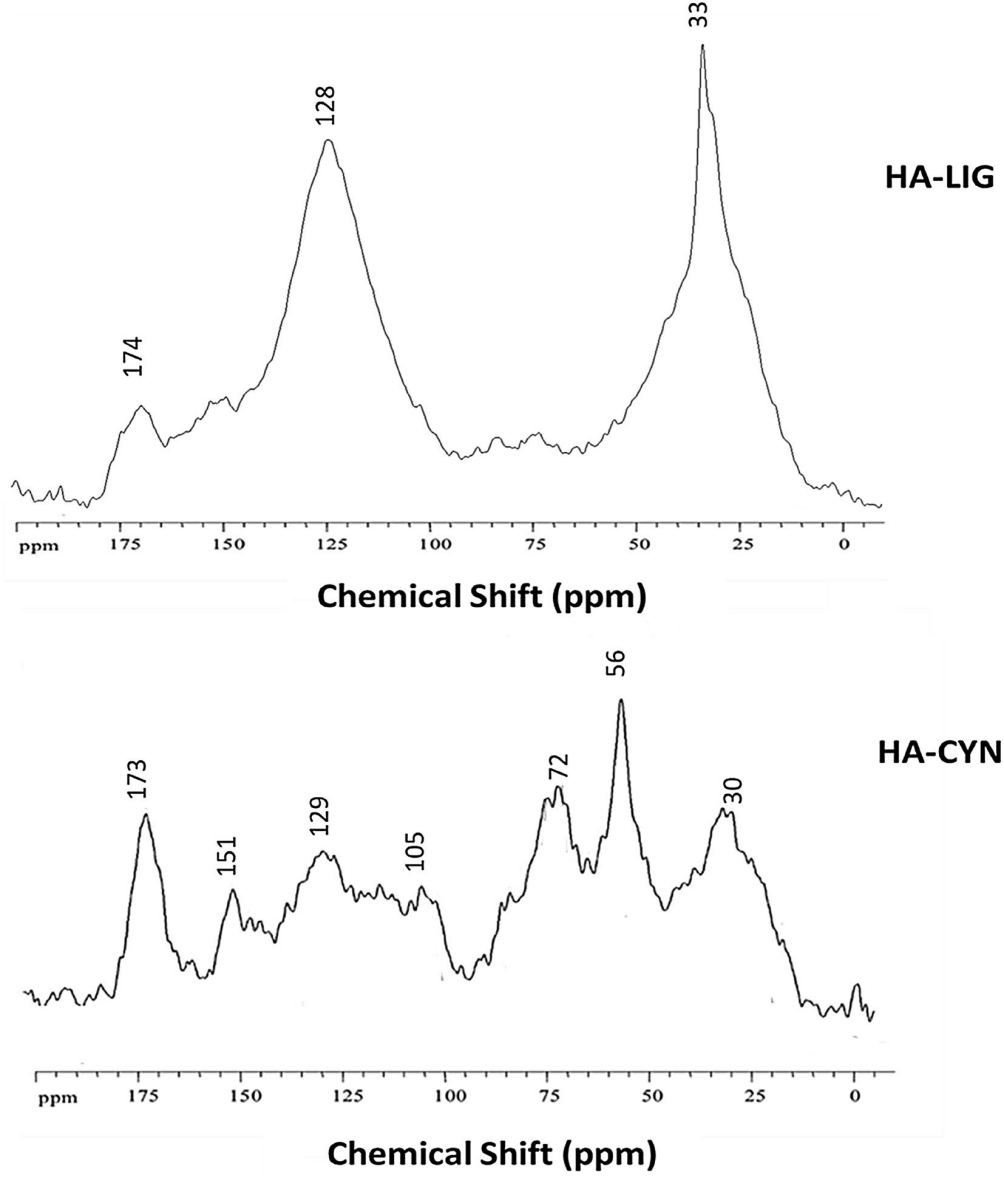
^13^C CPMAS NMR spectra of humic acids from lignite (HA-LIG) and artichoke biomasses composted (HA-CYN).

The ^13^C-CPMAS NMR spectrum of HA-LIG (Fig. 2) confirmed the predominance of apolar aliphatic and aromatic components which represented 28.7 and 42.2% of the total carbon content, respectively (Table 2). The two main resonances at 33 and 128 ppm indicated CH_2_ units in long alkyl chains and in C-substituted aromatic compounds, respectively, while the shoulder at 152 ppm is attributed to phenolic carbons (Fig. 2). A diversified C distribution among different functional groups was found in the solid-state NMR spectrum of HA-CYN (Table 2). The distinct signals around the broad resonance at 30 ppm in the 0–45 ppm range, are due to of methyl and methylene groups in alkyl chains of various linear lipid compounds, while the less intense peaks at 40–45 ppm may be due to tertiary (CH) and quaternary (C–R) carbons in assembled rings of sterol, flavonoids and lignans^25^. The prominent resonance at 57 ppm is mainly assigned to methoxy substituents in aromatic lignin monomers, with an additional contribution of C–N bonds of peptidic moieties^18,19^. The signals in the 60–110 ppm chemical shift pertain to various C–O groups in carbohydrates and polysaccharides from plant tissues. The resonance from 110 to 145 ppm is related to aromatic carbons, followed by the one of aryl C–O groups (145–160 ppm) in phenolic and lignin derivatives, which are commonly corresponding to resonances in the 145–60 ppm range^24^. The signal at 174 ppm is due to carboxyl carbons in different compounds, such as fatty acids, esters and peptides. The molecular properties of HA samples may be inferred by the dimensionless structural parameters such as aromaticity (ARM) and hydrophobicity (HB/HI) indexes and alkyl (A/OA), and lignin (LigR) ratios (Table 2), that are based on integration of spectral intervals^18,19,25^. The large values of HB/HI and ARM indexes and of A/OA ratio shown by the HA-LIG sample further highlight the large hydrophobic character provided to this material by the combined contribution of alkyl and aromatic structures. Conversely, less aromatic and alkyl carbon content, concomitant to an enhanced amount of C–O and C–N containing compounds, determined smaller values of HB/HI and A/OA for HA-CYN (Table 2). A different molecular composition of aryl C between the two HA was also suggested by the LigR ratio (Table 2), that resulted greater for HA-CYN, thus indicating a significant presence of lignin residues, also confirmed by the correlation between signals of methoxyl groups (45–60 ppm) and those of aryl C–O molecules (140–160 ppm), that agrees with the selective incorporation of lignin derivatives in HA-CYN revealed by thermochemolysis analyses^18,19,25^. Conversely, the decrease of LigR value in HA-LIG indicated a smaller content of the methoxyl groups of lignin molecules and a prevalence of phenol and polyphenolic components derived by the geochemical processes that formed the lignite from which HA-LIG was extracted.

**Table 2:** Relative contribution (%) and structural indices calculated from spectral data of main C structures over chemical shift regions (ppm) of ^13^C CPMAS-NMR spectra of HA-LIG and HA-CYN.

	Carboxyl-C	Phenol-C	Aryl-C	O-Alkyl-C	Methoxyl-C	Alkyl-C	Structural indexes			
	190–160	160–145	145–110	110–60	60–45	45–0	HB/HI^a^	A/OA^b^	ARM^c^	LigR^d^
HA-LIG	6.1	9.6	33.0	12.8	9.8	28.7	2.49	2.27	1.02	1.06
HA-CYN	10.9	5.7	20.0	27.5	13.8	22.2	0.92	0.81	0.52	2.43

^a^HB/HI = hydrophobicity index = [Σ (0–45) + (110–160)/ Σ (45–60) + (60–110) + (160–190)].

^b^A/OA = alkyl/O-alkyl ratio (0–45)/(60–10).

^c^ARM = aromaticity index [(110–160)/Σ (0–45) + (60–110)].

^d^LigR = Lignin ratio (45–60)/(140–160).

### ^1^H NMR spectra

**Figure 3:**
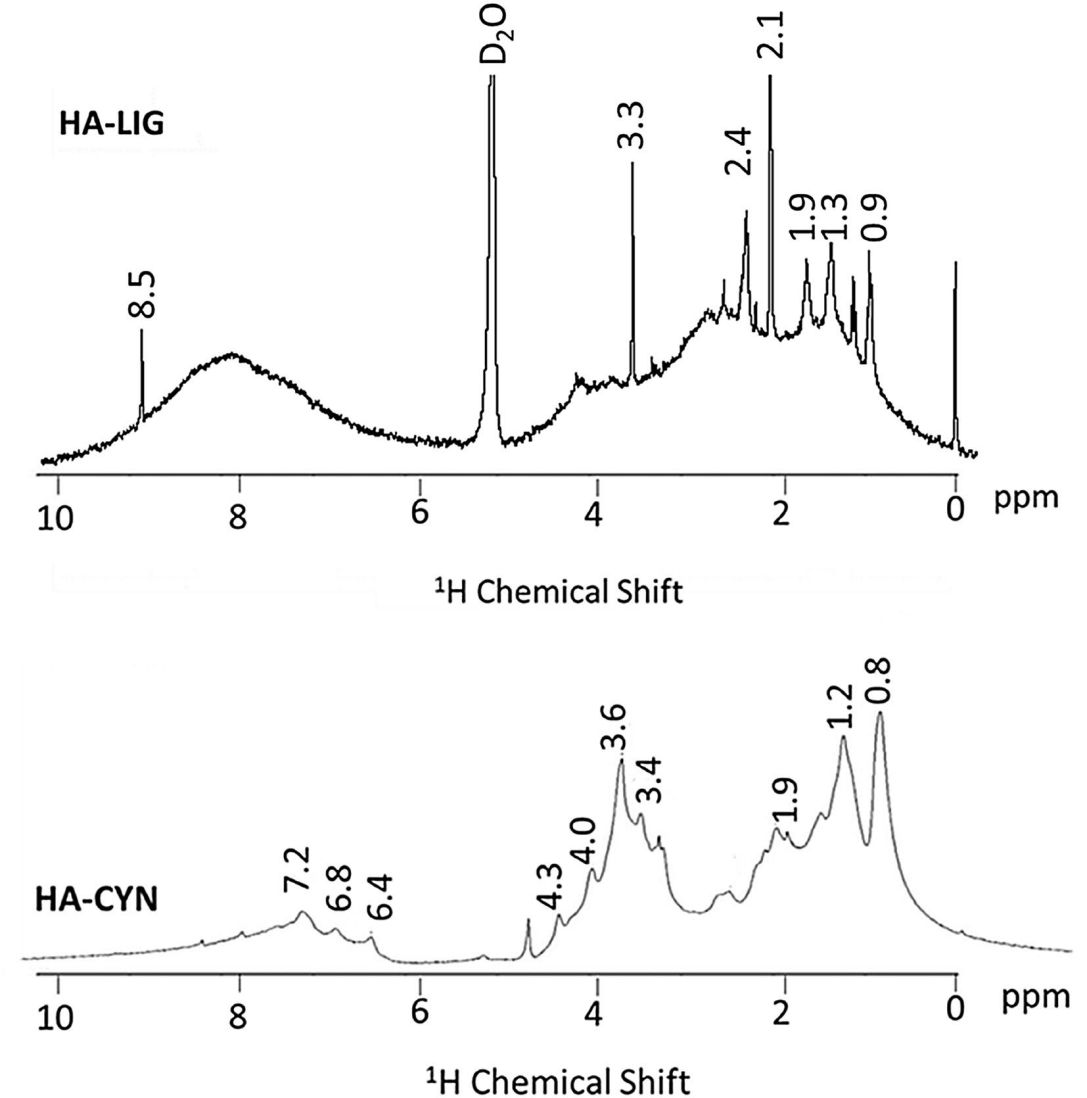
The liquid state ^1^H-NMR spectra of HA isolated from lignite (HA-LIG) and green compost made of artichoke residues (HA-CYN).

^1^H NMR spectra of the HA-LIG and HA-CYN (Fig. 3) can be divided into three main regions: 0–3 ppm, alkylic region (mostly aliphatics components); 3–5.5 ppm, O-Alkyl region (prevalently oligo- and polysaccharides); 6.5–8.5 ppm, aromatics region (mainly lignin components)^28,29^. The ^1^H NMR spectrum of HA-LIG shows several sharp signals emerging from the large resonance in the 0.9–2.4 ppm range, a single predominant signal at 3.3 ppm in the O-Alkyl region, and a conspicuous broad resonance in the aromatic and heterocyclic region over the 7–8.5 ppm range. The appearance of this spectrum is in line with the indications of the solid-state spectrum and confirms the large hydrophobicity of this humic matter of geochemical origin that contains prevalently alkyl and aromatic compounds. Conversely, the HA-CYN spectrum reveals a less intense resonance in both alkyl and aromatic region, but a greater signal intensity in the O-alkyl regions. Again, the features of the HA-CYN spectrum confirms the significantly smaller hydrophobicity of this material, as already noted for the CPMAS spectrum, and its richness in polar aliphatic compounds, such as the saccharidic and peptidic moieties derived from artichoke residues transformed in the green compost from which HA-CYN was isolated.

### High performance size exclusion chromatography (HPSEC) of HA

**Figure 4:**
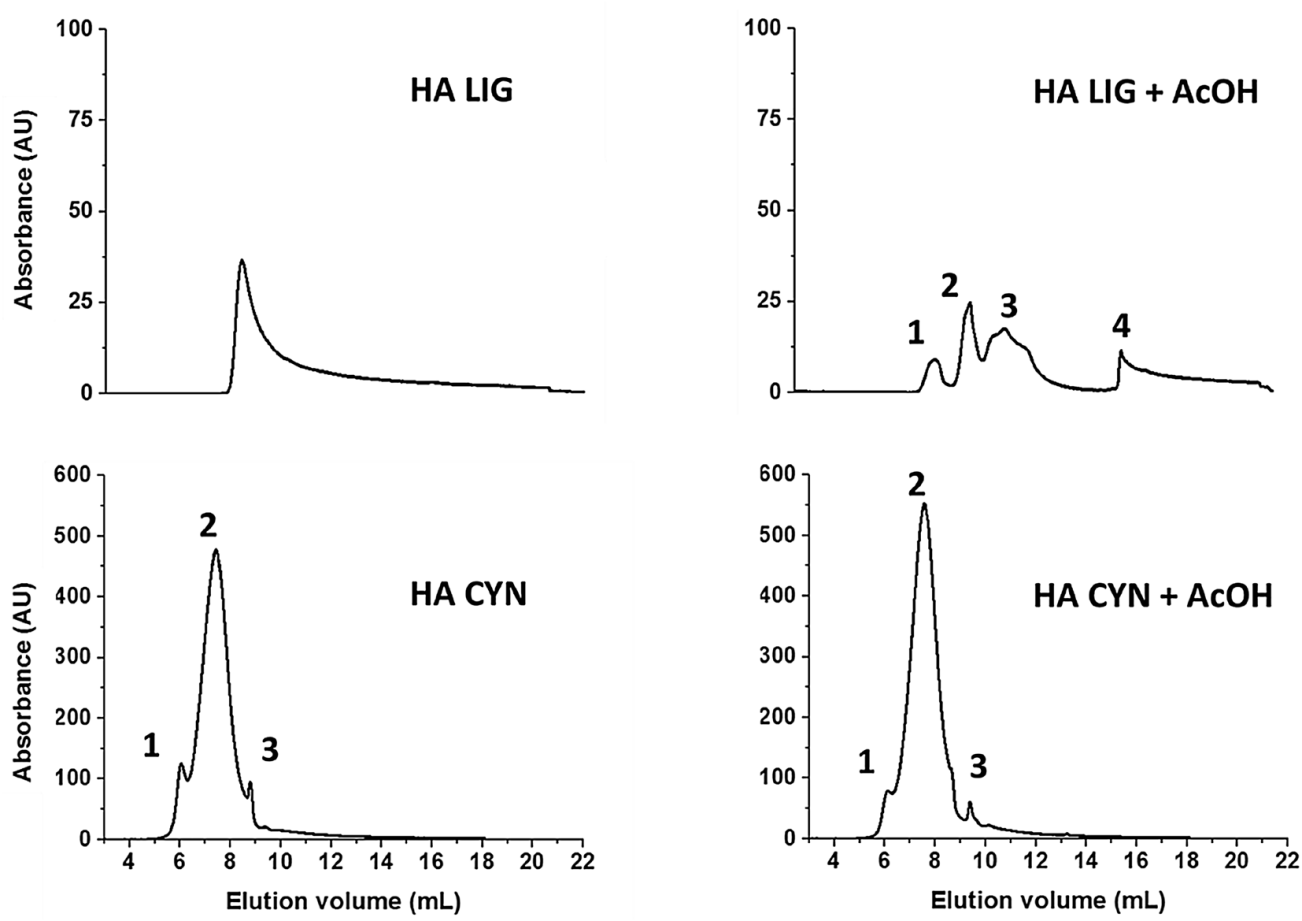
HPSEC chromatograms of HA-LIG and HA-CYN before and after addition of acetic acid (AcOH) to adjust sample pH from 7 to 3.5.

The HPSEC elution profiles before and after the treatment of humic solutions with acetic acid to lower the pH of the eluent from 7 to 3.5, has been used to evaluate the conformational structure of the loosely bound supramolecular associations of humic acids^30,31^. The chromatograms of both HA-LIG and HA-CYN before and after acetic acid additions are shown in Fig. 4. The two HA had a different HPSEC behaviour. In fact, while HA-LIG showed one single peak when injected at pH 7, the elution profile was disrupted in four different peaks after having adjusted the pH to 3.5 with AcOH before injection. Conversely, the elution profile of HA-CYN did not change between the two treatments revealing the same three peaks, although shifted to larger elution volumes due to the AcOH addition.

The different HPSEC behaviour of the two HA was reflected by the parameters calculated from the chromatograms (Table 3). For example, the Weight Average molecular weight (Mw) of HA-LIG was about 11,960 Da for the diffuse peak of the chromatogram of the material injected at pH 7, while the four peaks after pH adjustment to 3.5 revealed Mw of about 21,800, 10,370, 4240 and 180, respectively, passing from the one at smaller elution volumes, corresponding to material of greater molecular size, to that of lesser molecular size at larger elution volumes. Conversely, in the case of HA-CYN, the MW of the three peaks maintained similar values before and after AcOH addition, although the values observed for the latter treatment were lower than for former one, thereby agreeing with the noted peaks shift to larger elution volumes (Fig. 4). These results may be interpreted with the supramolecular understanding of humus from soil that describes the conformational structure of the HA as being stabilized by dispersive weak interactions among relatively small heterogeneous molecules, which may be disrupted by AcOH addition, due to formation of intermolecular hydrogen bonds stronger than the previous dispersive forces^26,30,31^. While this is confirmed for HA-LIG, whose material distribution in four peaks suggests a supramolecular association of smaller molecules, the same cannot be said for HA-CYN. In fact, the latter conformational arrangement appears to be mostly stabilized by covalent-bonded biopolymers, that survived the microbial composting process and are resilient to the disruption capacity of the AcOH treatment^32^. These may well be the polysaccharidic, peptidic, and polyphenolic lignin fragments, whose presence is indicated by FTIR, NMR and thermochemolysis results. Altogether, these findings indicate that HA-LIG was composed of relatively smaller compounds than HA-CYN, thereby conferring to the former material a greater conformational flexibility than the latter.

**Table 3:** Weight average (Mw) and number average (Mn) molecular weights, and polydispersity (P), as calculated from UV-detected HPSEC chromatograms for HA-LIG and HA-CYN, before and after addition of acetic acid (AcOH). Standard deviation was < 5%.

Sample	Peak interval (mL)	Mw	Mn	P
HA LIG	7.5–18.0	11,957	1774	6.7
HA LIG + AcOH-peak 1	7.5–9.2	21,792	21,314	1.0
HA LIG + AcOH-peak 2	9.2–10.5	10,374	10,172	1.0
HA LIG + AcOH-peak 3	10.5–14.9	4238	3396	1.2
HA LIG + AcOH-peak 4	15.2–22.3	180	87	2.1
HA CYN-peak 1	4.7–6.3	86,258	85,187	1.0
HA CYN-peak 2	6.3–8.7	41,437	38,391	1.1
HA CYN-peak 3	8.7–9.3	18,489	18,412	1.0
HA CYN + AcOH-peak 1	4.7–6.3	84,536	83,560	1.0
HA CYN + AcOH-peak 2	6.3–8.7	37,934	34,658	1.1
HA CYN + AcOH-peak 3	9.2–9.6	13,783	13,724	1.0

### Antiflammatory activity of HA: in vitro assays on HaCaT cells

#### Cells viability

Cell viability was assessed by the Trypan blue method with different increasing concentrations of HA-LIG and HA-CYN. The treatment of human keratinocyte cells with both humic extracts did not influence cells viability despite the maximum applied concentration (Figure S2). Contrary to earlier works^33,34^, these results exclude a possible cytotoxic activity of these HA on human cells cultures. Additionally, HaCaT cells pretreated with both HA at the same concentration (50 µg mL^−1^) showed a statistically significant viability increase, as compared to the cells treated with only Urban Dust (Figure S2). In fact, the cell viability after application of HA-CYN and HA-LIG was still 87% and 82% of that of the untreated control, respectively, whereas the inflammatory stimulation by Urban Dust decreased viability of HaCaT cells by 62%, as also shown by previous studies^35^. These results suggest a protective effect by humic matter towards human cells survival. A similar behaviour was reported by fulvic acids which apparently protected the activity of RAW 264.7 human cells cultures^36^.

The capacity of natural organic matter to protect the activity of human cells is an important prerequisite for the development of novel and sustainable antioxidant systems^37^. In fact, an oxidative cell stress typically indicates that the balance between ROS and the antioxidant system is compromised^38^, thus fostering an increase in cells free-radicals content that causes oxidative damages, such as DNA modification, and lipid oxidation or peroxidation^39^. It has been suggested that the antioxidant and protective role of humic materials may be related to their redox properties^40^, which may inactivate reactive oxygen species by their binding to the humic structure through a reversible and reproducible process^41^. This behaviour may well explain our results indicating that addition of humic matter induces a normal proliferation of HaCaT, cells despite the Urban Dust treatment^42^. However, no univocal structure–activity relationship was yet produced to explain the molecular bases of HA bioactivity against human diseases. In this work, the protection properties of HA-CYN are related to the large content of phenolic and lignin components and in accordance to previous studies on phenolic materials, in which mono- and oligo-hydroxylated aromatic units were shown to exhibit protective activities^18,19,43^. Similarly, the protection activity of HA-LIG is be related to its hydrophobic composition as reported in both NMR and thermochemolysis results (Tables 2, S1, S2; Figs. 1, 3). While previous studies pointed out that the antimicrobial activities promoted by humic and Humic‐like substances (HULIS) against plants pathogens depended on their specific molecular composition^18,19,44,45^, we show for the first time that HA isolated from composted agricultural biomasses exert protection properties on human cells and this depends on the molecular composition of the tested humic acid.

#### Effects of HA-LIG and HA-CYN on IL-1β and IL-6 gene expression after Urban Dust exposure

The keratinocyte model is commonly used to evaluate the effects of irritants in dermatology due the presence of these cells in the outer layers of the skin and their preliminary role in the encounter with foreign irritants^46^. Moreover, keratinocytes play a crucial role to maintain the barrier of the stratum corneum that protects the inner layers of skin cells^47^. Additionally, keratinocytes can produce a wide range of inflammatory mediators which further induce the upregulation and secretion of secondary mediators, including chemokines, resulting in the recruitment of leukocytes towards the damaged area of ​​the skin^48^.

It has been reported that fine dust (FD) elicited inflammatory responses in keratinocytes and transferred inflammatory responses to macrophages possibly mediated by the proinflammatory cytokine Interleukin-1β (IL-1β)^49^. IL-1β is a potent pro-inflammatory cytokine that is crucial for host-defence responses to infection and injury produced and secreted by the innate immune system cells, such as monocytes and macrophages^50^. It is produced without a signal sequence and does not follow the conventional route of protein secretion, but rather employs one or more non-conventional pathways of secretion^51^. Conversely, interleukin-6 (IL-6) is produced transiently in response to infection during its acute phase. The continuous dysregulated synthesis of IL-6 induced a pathological effect such as chronic inflammation and autoimmunity, although its normal expression is tightly controlled by transcriptional and post-transcriptional mechanisms^52^. Furthermore, IL-6 plays a crucial role in inducing an epithelial response to environmental inputs^35^.

**Figure 5:**
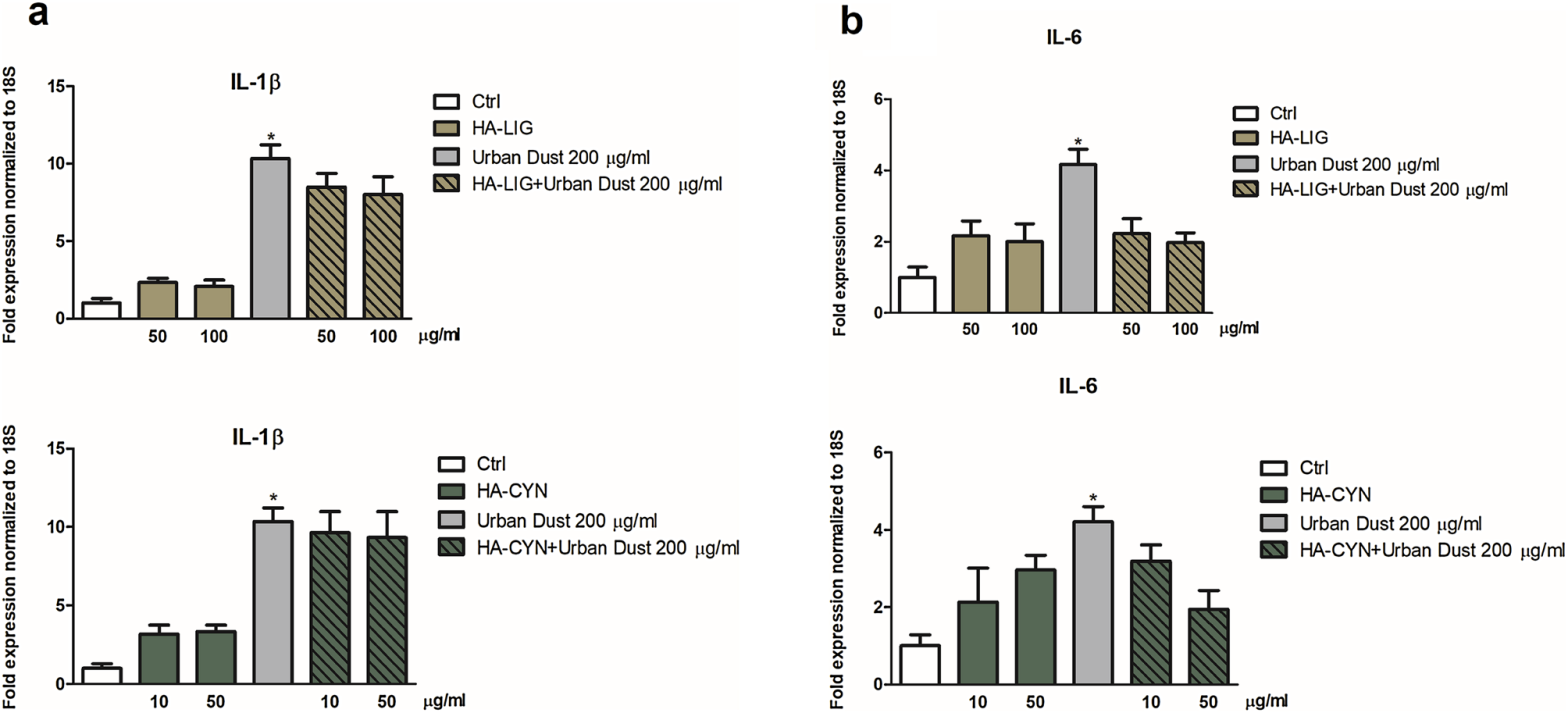
Cellular IL-1β (**a**) and IL-6 (**b**) levels were measured in pellets from HaCaT cells pre-treated for 2 h with either HA-LIG or HA-CYN, and then added with 200 µg mL^−1^ of Urban Dust, versus a first control of untreated cells (Ctrl), and a second control of cells treated only with 200 µg mL^−1^ of Urban Dust. Statistical significance of Urban Dust-treated cells was determined in respect to the 100% of IL-1β and IL-6 rate in untreated control cells (*). Data were expressed as mean ± SD of three independent experiments, each performed in triplicate. Student’s t-test was used to calculate significant differences (*P < 0.05).

In this work, the gene expression of IL-1β and IL-6 was assessed to verify the effective antiflammatory efficacy of HA-CYN and HA-LIG on HaCaT cells treated with Urban Dust, as suggested by preliminary viability experiments (Fig. 5). Our results revealed a significant increase of IL-1β and IL-6 gene expression after the stimulation by Urban Dust (Fig. 5a,b). Conversely, an application of Urban Dust on HaCaT cells after their treatment with either HA-LIG or HA-CYN induced a decrease of cytokines expression (Fig. 5a,b). HA-LIG was more effective than HA-CYN by inducing a less IL-1β expression, whereas the HA-CYN treatment exhibited a slightly larger anti-flammatory activity in the case of IL-6. Moreover, the analysis of markers levels of the inflammatory process in HaCaT cells suggested the beneficial effects of both humic extracts to reduce the inflammation induced by Urban Dust (Fig. 5a,b).

These results are in accordance with previous studies that reported an inhibitory effect of humic derivates on cytokines release associated with an in vitro^22,53^. The biochemical explanation of antiflammatory effects of HA was related to the inhibition of the 5-lipoxygenase pathway of the arachidonic acid (AA) cascade by naturally occurring HA as well as synthetic HA-like polymers^53^. Moreover, the 65–90% suppression of the heat-induced AA release by either natural sodium humates or humic-like products obtained by caffeic acid oxidation (KOP) supported the characterization of HA as antiflammatory materials^22^. On the other hand, it has been found that humic substances stimulated the release of pro-inflammatory cytokines, such as TNF-α in vitro, but only in the presence of exogenous lipopolysaccharides, thus suggesting that humic substances alone do not cause inflammation under normal conditions^54^. Additionally, potassium humates were reported to induce a positive effect on the proliferation of lymphocytes cells^20^. In this case, cells proliferation was increased as a response to autocrine growth pathway characterized by synthesis of cytokines and expression of T cell surface receptors for these mediators, as well as by the production of the primary autocrine growth factor, such as IL-2, and the enhanced expression of the IL-2 receptor (CD25)^55^.

Our findings represent an innovative approach to relate the effect on HaCaT cells to the molecular composition of humic matter.

Several studies pointed out the occurrence of beneficial effects of humic matter, also from composted biomasses, on either plants or microorganisms at molecular, physiological and transcriptional levels^56^. A working hypothesis of the bioactive effects of humic matter relies on the concomitant combination of conformational structure and molecular composition on the interaction with a different type of cells^57^. In fact, the difference between HA-CYN and HA-LIG in antiflammatory activities on the treated HaCaT cells is explained by the different molecular and conformational properties of the employed HA, as indicated by NMR, thermochemolysis and HPSEC results. The inhibition of the release of IL-6 and IL-1β inflammatory cytokine by HA-LIG when added at 50 µg mL^−1^ before Urban Dust may be related to its dominant hydrophobic properties (large HB/HI index), whereas the slighter inhibition of the IL-1β cytokine by HA-CYN, at the same concentration of HA-LIG, may be accounted to its large content of polyphenolic lignin components.

The different conformational stability of HA revealed by the changes in HPSEC elution profiles after AcOH addition may also represent an additional factor that contributes to the antiflammatory behavior of HA (Table 3, Fig. 4). However, in the case of cells viability (Figure S2), both HA-LIG and HA-CYN showed the same positive protective effect, despite the former was found to be composed of smaller molecular associations than the latter. This indicates that the HA molecular composition, rather than the conformational stability, is responsible for the effectiveness of the protection of cells viability. In particular, it is the content of hydrophobic components in HA-LIG, as shown by the large values of HB/HI, ARM and A/OA indexes, and the amount of lignin-derived polyphenols in HA-CYN, as revealed by the great LigR ratio (Table 2), to make both materials effective in cell protection. This finding is in line with previous studies in which phenolic components were shown to suppress human chronic diseases, and with other ones that reported that the hydrophobicity and phenolic content, rather than molecular size, stimulated the activity of plant root cells. Conversely, the greater effect of HA-LIG in the suppression of the expression of both IL-1β and IL-6 cytokines (Fig. 5a,b) may be explained by the lesser conformational stability of this material than HA-CYN that exhibits a more rigid conformational structure. In fact, the HA-LIG conformation could be disrupted into smaller humic superstructures by interacting with HaCaT cells and concomitantly release small bioactive phenolic molecules that significantly reduces the inflammation. Conversely, in the case of HA-CYN, its observed conformational stability prevents a similar break down in smaller aggregates and a release of bioactive molecules as easily as for HA-LIG, thereby showing a lesser cytokines suppression in HaCaT cells treated with Urban Dust.

### Limitations of study

The aim of this study was to investigate the anti-inflammatory properties of two characterized humic acids (HAs) extracted from two natural sources. Despite the molecular differences between HA were extensively explained, we hypothesize that a possible limitation of this work consists in having used only the humic acid fraction of compost humus, while fulvic acids alone or a mixture of the two (HA and FA) may reveal different anti-inflammatory processes. However, for this very reason, the observed relationship between molecular structure and biological activity of HA assumes an even greater role in our work since it describes a specific anti-inflammatory activity of the HA fraction.

An additional shortcoming of our work may be due to have evaluated the anti-inflammatory activity on a single cell line and quantified a limited number of key inflammatory cytokines. Our preferential selection of IL-6 and IL-1β as two inflammatory markers was based on previous studies that have shown their role as highly sensitive molecules of the cytokine pathway to the inflammatory stimulation^58,59^, thereby justifying their choice as preliminary biomarkers of humic matter bioactivity.

These considerations imply that further studies are required to explore in greater details the beneficial properties of humic materials in medical applications^60^ and identify the molecular and cellular mechanisms underpinning their anti-inflammatory proprieties. Nevertheless, we believe in the pioneering validity of our study since our unprecedented results can stimulate further investigations of the anti-inflammatory activity of well characterized humic matter on human keratinocytes.

## Conclusions

Medical applications of humic substances are related to the anti-viral, antiflammatory, antioxidant, anti-tumoral and anti-toxin proprieties of these materials. Our findings showed antiflammatory activities of two different HA isolated from either a non-renewable geochemical deposit, such as lignite, or a renewable and sustainable source like green compost. Their difference in molecular compositions was evaluated by FTIR spectrometry, NMR spectroscopy, off-line thermochemolysis associated to GC–MS, while their conformational structure was assessed by HPSEC. Our results from specific assays suggest that HA antiflammatory activities on HaCaT cells were partially related to their hydrophobic properties conferred by aromatic compounds and polyphenolic lignin fragments and partially on the conformational stability of their supramolecular associations.

This work innovatively evaluated HA bioactivity on a keratinocyte model after stimulation by Urban Dust and on the consequent HaCaT cellular production of IL-1β and IL-6 cytokines, by attempting a relation between the HA molecular properties and their observed bioactivities. It is suggested that HA bioactive properties are due not only to their degree of hydrophobicity that promotes their adhesion on the surface of the target cells and increase general cells viability, but also to the conformational arrangement of their molecular complexity. We found that HA associated in less stable conformation may facilitate the release of bioactive components when in interactions with cells and decrease cellular expression of inflammatory cytokines. We believe that this preliminary identification of the necessary element to draw a structure–activity relationship between well characterized HA and their bioactivity represents an important step forward in supporting the potential exploitation of natural sustainable materials in the dermatological field.

## Materials and methods

### Extraction of humic acids (HA)

Humic acids were extracted from both a North Dakota lignite (HA-LIG) and a green compost made by artichoke residues (HA-CYN)^18,19^. The green compost was produced in the composting facility of the Experimental Farm of the University of Napoli Federico II at Castel-Volturno (CE) and was obtained by mixing residues of artichoke production with woodchips from poplar trimming at 70/30 w/w ratio. The on-farm composting processes were performed on static piles spread on an air insufflation system formed by perforated rubber tubes and a rotative pump. The composting processes lasted 100 days with an initial 45 days of active phase where the pile temperature reached about 70 °C. At the end of composting process, the piles were randomly sampled to obtain a final amount of 1 kg for each compost. The organic materials were air dried, sieved at 2 mm and stored a 4 °C until further analyses.

HA were obtained by suspending 100 g of humic matter (artichoke compost and North Dakota lignite, respectively) in 1000 mL of 1 M KOH solution and mechanically shaking the mixture for 24 h. The suspension was then centrifuged at 7000 rpm for 20 min and the supernatant filtered through glass-wool. The extraction was repeated twice. The combined extracts were acidified to pH 1 with 6 M HCl and allowed to settle for 24 h. Then, the samples were centrifuged at 4000 rpm for 20 min and the HA residues dialyzed (1kD cutoff Spectrapore membranes) against deionized water until Cl-free. The HA were then freeze-dried for further analytical characterization.

### Elemental analyses and FTIR–ATR spectroscopy of HA

The elemental composition of HA-LIG and HA-CYN was determined with a Fison EA 1108 Elemental Analyzer. Infrared (IR) spectra were recorded on a Perkin–Elmer Frontier Fourier transform infrared spectrometer using an attenuated total reflection (ATR) device equipped with a diamond/ZnSe crystal. About 2 mg of finely ground powder was inserted in the crystal device and the contact was obtained applying on sample a strength of about 150 N. Each spectrum was subjected to 32 scans with the resolution of 4 cm^−1^ in the 4000–400 cm^−1^ region.

### Offline pyrolysis TMAH-GC–MS of HA

HA samples (500 mg) was placed in a quartz boat and moistened with 1 mL of TMAH (25% in methanol) solution. After drying the mixture under a gentle stream of nitrogen, the quartz boat was introduced into a Pyrex tubular reactor (50 cm × 3.5 cm i.d.) and heated at 400 °C for 30 min in a furnace (Barnstead Thermolyne 21,100). The products released by thermochemolysis were transferred online by a helium flow (20 mL min^−1^) into a series of two chloroform (50 mL) traps kept in ice/salt baths. The extracts were concentrated by rota-evaporation. The residue was resuspended in 1 mL of chloroform and transferred in a glass vial for GC–MS analysis. The GC–MS analyses were performed with a Perkin- Elmer Autosystem XL by using a RTX-5MS WCOT capillary column, (Restek, 30 m × 0.25 mm; film thickness, 0.25 μm) that was coupled, through a heated transfer line (250 °C), to a PE Turbomass-Gold quadrupole mass spectrometer. The chromatographic separation was carried out with the following program: 60 °C (1 min isothermal), rate 7 °C min^−1^ to 320 °C (10 min isothermal). Helium was applied as carrier gas at 1.60 mL min^−1^, the injector temperature was at 250 °C, the split-injection mode had a 30 mL min^−1^ of split flow. Mass spectra were obtained in EI mode (70 eV), scanning in the range 45–650 m/z, with a cycle time of 1 s. Compound identification was based on comparison of mass spectra with the NIST library database, previous published spectra and standard.

### NMR spectroscopy of HA

A 300 MHz Bruker Avance spectrometer, equipped with a 4 mm wide-bore MAS probe, was used to obtain solid-state HA spectra. Each fine-powdered sample (5 mg) was packed into a 4 mm zirconium rotor, provided with a Kel-F cap, and spun at a rate of 13,000 ± 1 Hz. The ^13^C NMR spectra were acquired through Cross-Polarization Magic-Angle-Spinning (CPMAS) technique by using 2 s of recycle delay, 1 ms of contact time, 30 ms of acquisition time and 4000 scans. Liquid-state NMR spectra of HA were performed on a 400-MHz Bruker Avance spectrometer, equipped with a 5-mm Bruker BBI (Broad Band Inverse) probe. Each sample (5.0 mg mL^−1^) was dissolved with deuterated water and placed into a 5.0-mm quartz tube. ^1^H NMR spectra were acquired with 2 s of thermal equilibrium delay, 90° pulse length ranging within 8.5 and 9.5 µs, 32,768 time-domain points, and 64 transients.

### High performance size exclusion chromatography

The HPSEC system consisted of a Shimadzu LC-10-AD pump equipped with a Rheodyne rotary injector and 100-µL sample loop and a UV/VIS detector (Perkin e Elmer LC295), set at 280 nm. A PolySep™ GFC-P3000 300 × 7.80 mm (Phenomenex, USA) was employed, and it was preceded by a PolySep GFC-P 35 × 7.80 safety guard (Phenomenex, USA) and a 2 mm inlet filter. The elution flow rate was set to 0.6 mL min^−1^, whereas the eluent was made of 0.1 mol L^−1^ NaH_2_PO_4_ solution (buffered at pH 7.0) added with 4.6 mmol L^−1^ NaN_3_. Both mobile phase and HA solutions were filtered through 0.45 μm Millipore filter prior to the chromatographic analyses. Column calibration was carried out by using sodium polystyrene sulfonates of known molecular masses: 123,000, 16,900 and 6780 Da. Furthermore, ferulic acid (194 Da) and catechol (110 Da) were used as low molecular weight standards. Humic acids were solubilised in the eluent solution at a concentration of 0.6 g L^−1^ and eluted by HPSEC. The same humic solutions were then added with glacial acetic acid (AcOH) to lower their pH to 3.5 and injected again into the HPSEC system. The obtained relations between molar mass (MM) and elution volume (EV) were: log MM = 0.1407 × EV + 6.4077 (R^2^ = 0.996). Weight Average (Mw) and Number Average (Mn) molecular weights and polydispersity (P) were calculated. A Unipoint Gilson Software was used to record and elaborate the chromatograms, while the calculations of Mw and P were performed by the Origin software (v. 9.1, Originlab).

### Anti-iflammatory activity

#### Cell culture

Immortalized human keratinocytes, HaCaT cells, were grown in Dulbecco’s modified Eagle’s medium (DMEM, GIBCO, Grand Island, NY) containing 10% fetal bovine serum (FBS, GIBCO, Grand Island, NY), 2 mM l-glutamine (GIBCO, Grand Island, NY) and antibiotics (100 IU mL^−1^ penicillin G, 100 μg mL^−1^ streptomycin, GIBCO, Grand Island, NY, USA). Cells were cultured in a humidified incubator at 37 °C with 5% CO_2_.

#### Chemicals

Urban Dust (SRM 1649b), supplied by NIST (Gaithersburg, MD, USA), was dissolved in phosphate-buffered saline 1X (PBS, GIBCO, Grand Island, NY, USA), sonicated for 30 min to avoid agglomeration and diluted in DMEM to obtain the treating concentration of 200 μg mL^−1^. HA-LIG and HA-CIN extracts were dissolved in PBS and diluted in DMEM to obtain the treating concentration of 50 and 100 μg mL^−1^ for HA-LIG and of 10 and 50 μg mL^−1^ for HA-CIN.

### Treatment

HaCaT cells were plated onto 60 mm culture plates in 3 mL of fresh culture medium until they reached a confluence of about 70%. Cells were covered with DMEM and pre-incubated for 2 h with HA-LIG (50 and 100 µg mL^−1^) and HA-CYN (10 and 50 µg mL^−1^) purified extracts, before the treatment with Urban Dust 1649b at a concentration of 200 μg mL^−1^ for 4 h.

#### Analysis of cell viability

Six hours after treatment, cell viability was determined with the Trypan blue method. Cells were washed twice with PBS, incubated with trypsin/EDTA for 5 min and centrifuged at 1000×*g* for 10 min. The cell pellet was resuspended in an appropriate volume of PBS, and 10 µL of the cell suspension was combined with 10 µL of Trypan blue solution. The mix was incubated for 15 min at room temperature and the number of unstained cells (vital cells) and the total number of cells (vital and not) were determined on the hemacytometer under a microscope (dead cells would take up the Trypan blue stain). The percentage of viable cells was determined as the number of unstained cells divided by the total number of cells.

#### RNA extraction and real-time

Treated HaCaT cells were evaluated for their Cytokine levels. Total mRNA was isolated using the RNeasy Mini Kit (Qiagen, Doncaster, Australia) according to the manufacturer’s instructions. cDNA was prepared using the Transcriptor High fidelity cDNA Synthesis Kit (Roche, Indianapolis, IN, USA). Quantitative reverse transcriptase polymerase chain reaction (qRT-PCR; LightCycler, Roche, Indianapolis, IN, USA) was performed to assess gene expression of IL-6 and IL-1β. PCR primers were designed based on published sequences, and their specificity was verified with BLAST alignment search. The amount of mRNA for a given gene in each sample was normalized to the amount of mRNA of 18S reference gene in the same sample.

### Statistics

All statistical analyses were performed using GraphPad Prism 4.0 (GraphPad Software Inc, La Jolla, CA, USA). Data that passed the normality test were analysed with two-tailed, t test. Values of P < 0.05 were considered significant.

## Supplementary Information

Supplementary Information.

## Data Availability

All data generated or analysed during this study are included in this published article or in the accompanying Supplementary Information files.

## References

[1] Adame JA, Notario A, Villanueva F, Albaladejo J (2012). Application of cluster analysis to surface ozone, NO_2_ and SO_2_ daily patterns in an industrial area in Central-Southern Spain measured with a DOAS system. Sci. Total Environ..

[2] WHO (2014). 7 million deaths annually linked to air pollution. Cent. Eur. J. Public Health.

[3] Manisalidis I, Stavropoulou E, Stavropoulos A, Bezirtzoglou E (2020). Environmental and health impacts of air pollution: A review. Front. Public Health..

[4] Babadjouni RM, Hodism DM, Radwanski R, Durazo R, Patel A, Liu Q, Mack WJ (2017). Clinical effects of air pollution on the central nervous system; a review. J. Clin. Neurosci..

[5] Guo H, Chang Z, Wu J, Li W (2019). Air pollution and lung cancer incidence in China: Who are faced with a greater effect?. Environ. Int..

[6] Baek JO, Cho J, Roh JY (2021). Associations between ambient air pollution and medical care visits for atopic dermatitis. Environ. Res..

[7] Farage MA, Katsarou A, Maibach HI (2006). Sensory, clinical and physiological factors in sensitive skin: A review. Contact Dermatitis.

[8] Abolhasani R, Araghi F, Tabary M, Aryannejad A, Mashinchi B, Robati RM (2021). The impact of air pollution on skin and related disorders: A comprehensive review. Dermatol. Ther..

[9] Wang J, Li Y, Zhao P, Tian Y, Liu X, He H, Jia R, Oliver BG, Li J (2020). Exposure to air pollution exacerbates inflammation in rats with preexisting COPD. Mediat. Inflamm..

[10] Ferrara F, Prieux R, Woodby B, Valacchi G (2021). Inflammasome activation in pollution-induced skin conditions. Plast. Reconstr. Surg..

[11] Rembiesa J, Ruzgas T, Engblom J, Holefors A (2018). The impact of pollution on skin and proper efficacy testing for anti-pollution claims. Cosmetics.

[12] Cordell GA (2009). Sustainable drugs and global health care. Quim. Nova.

[13] Fabbrocini G, Staibano S, De Rosa G, Battimiello V, Fardella N, Ilardi G, La Rotonda MI, Longobardi A, Mazzella M, Siano M, Pastore F, De Vita V, Vecchione ML, Ayala F (2011). Resveratrol-containing gel for the treatment of acne vulgaris: A single-blind, vehicle-controlled, pilot study. Am. J. Clin. Dermatol..

[14] Lembo S, Balato A, Di Caprio R, Cirillo T, Giannini V, Gasparri F, Monfrecola G (2014). The modulatory effect of ellagic acid and rosmarinic acid on ultraviolet-B-induced cytokine/chemokine gene expression in skin keratinocyte (HaCaT) cells. BioMed. Res. Int..

[15] Di Caprio R, Monfrecola G, Gasparri F, Micillo R, Balato A, Lembo S (2020). Milk thistle and olive extract: Old substances with a new mission against sun-induced skin damage. Giornale Ital. Dermatol. Venereol..

[16] Varga Z, Rafay S, Sabzwari A, Vargova V (2017). Cardiovascular risk of nonsteroidal antiflammatory drugs: An under-recognized public health issue. Cureus.

[17] Klöcking R, Helbig B (2001). Humic substances, medical aspects and applications. Biopolym. Online.

[18] Verrillo M, Salzano M, Cozzolino V, Spaccini R, Piccolo A (2021). Bioactivity and antimicrobial properties of chemically characterized compost teas from different green composts. Waste Manage..

[19] Verrillo M, Cozzolino V, Spaccini R, Piccolo A (2021). Humic substances from green compost increase bioactivity and antibacterial properties of essential oils in Basil leaves. Chem. Biol. Technol. Agric..

[20] Jooné GK, Dekker J, van Rensburg CE (2003). Investigation of the immunostimulatory properties of oxihumate. J. Biosci..

[21] Van Rensburg CEJ (2015). The antiinflammatory properties of humic substances: A mini review. Phytother. Res..

[22] Jansen Van Rensburg CE, Naude PJ (2009). Potassium humate inhibits complement activation and the production of inflammatory cytokines in vitro. Inflammation.

[23] Nuzzo A, Buurman P, Cozzolino V, Spaccini R, Piccolo A (2020). Infrared spectra of soil organic matter under a primary vegetation sequence. Chem. Biol. Technol. Agric..

[24] Savy D, Cozzolino V, Vinci G, Nebbioso A, Piccolo A (2015). Water-soluble lignins from different bioenergy crops stimulate the early development of maize (*Zea mays* L.). Molecules.

[25] Monda H, Cozzolino V, Vinci G, Spaccini R, Piccolo A (2017). Molecular characteristics of water-extractable organic matter from different composted biomasses and their effects on seed germination and early growth of maize. Sci. Total Environ..

[26] Piccolo A, Conte P, Trivellone E, Van Lagen B, Buurman P (2002). Reduced heterogeneity of a lignite humic acid by preparative HPSEC following interaction with an organic acid. Characterization of size-separates by Pyr-GC-MS and 1H-NMR spectroscopy. Environ. Sci. Technol..

[27] Deport C, Lemée L, Amblès A (2006). Comparison between humic substances from soil and peats using TMAH and TEAAc thermochemolysis. Org. Geochem..

[28] Simpson AJ, McNally DJ, Simpson MJ (2011). NMR spectroscopy in environmental research: From molecular interactions to global processes. Prog. Nucl. Magn. Reson. Spectrosc..

[29] Sun J, Drosos M, Mazzei P, Savy D, Todisco D, Vinci G, Pan G, Piccolo A (2017). The molecular properties of biochar carbon released in dilute acidic solution and its effects on maize seed germination. Sci. Total Environ..

[30] Piccolo A, Conte P, Cozzolino A (1999). Effects of mineral and monocarboxylic acids on the molecular association of dissolved humic substances. Eur. J. Soil Sci..

[31] Piccolo A (2002). The supramolecular structure of humic substances: A novel understanding of humus chemistry and implications in soil science. Adv. Agron..

[32] Piccolo A, Conte P, Cozzolino A (2001). Chromatographic and spectrophotometric properties of dissolved humic substances compared with macromolecular polymers. Soil Sci..

[33] Yang HL, Lu FJ, Wung SL, Chiu HC (1994). Humic acid induces expression of tissue factor by cultured endothelial cells: Regulation by cytosolic calcium and protein kinase C. Thromb. Haemost..

[34] Hseu YC (2008). Humic acid induced genotoxicity in human peripheral blood lymphocytes using comet and sister chromatid exchange assay. J. Hazard. Mater..

[35] Liu CW (2018). PM2.5-induced oxidative stress increases intercellular adhesion molecule-1 expression in lung epithelial cells through the IL-6/AKT/STAT3/NF-κB-dependent pathway. Fibre Toxicol..

[36] Jayasooriya RGPT, Dilshara MG, Kang CH, Lee S, Choi YH, Jeong YK, Kim GY (2016). Fulvic acid promotes extracellular anti-cancer mediators from RAW 264.7 cells, causing to cancer cell death in vitro. Int. Immunopharmacol..

[37] Efimova IV, Khilko SL, Smirnova OV (2012). Antioxidant activity of humic acids in radical-chain oxidation processes. Russ. J. Appl. Chem..

[38] Poljsak B, Šuput D, Milisav I (2013). Achieving the balance between ROS and antioxidants: When to use the synthetic antioxidants. Oxid. Med. Cell. Longev..

[39] Barrera G (2012). Oxidative stress and lipid peroxidation products in cancer progression and therapy. ISRN Oncol..

[40] Luciani G, Venezia V, Silvestri B, Vitiello G, Di Donato P, Landi G, Mollo V, Verrillo M, Cangemi S, Piccolo A (2022). Chemical squeezing of humic acids in hybrid SiO_2_ nanostructures tunes their antioxidant properties. Chemosphere.

[41] Zykova MV, Schepetkin IA, Belousov MV, Krivoshchekov SV, Logvinova LA, Bratishko KA, Yusubov MS, Romanenko SV, Quinn MT (2018). Physicochemical characterization and antioxidant activity of humic acids isolated from peat of various origins. Molecules.

[42] Szot K, Góralczyk K, Michalska M, Veryho N, Chojnowski J, Ponikowska I, Rość D (2019). The effects of humic water on endothelial cells under hyperglycemic conditions: Inflammation-associated parameters. Environ. Geochem. Health.

[43] Vuolo MM, Lima VS, Maróstica Junior MR (2018). Phenolic compounds: Structure, classification, and antioxidant power. Bioact. Compd. Health Benefits Potential Appl..

[44] Pane C, Spaccini R, Piccolo A, Celano G, Zaccardelli M (2019). Disease suppressiveness of agricultural greenwaste composts as related to chemical and bio-based properties shaped by different on-farm composting methods. Biol. Control.

[45] Verrillo M, Savy D, Savarese C, Cangemi S, Cozzolino V, Piccolo A (2021). Valorization of lignins from energy crops and agro-industrial byproducts as antioxidant and antimicrobial materials. J. Sci. Food Agric..

[46] Nguyen AV, Athena MS (1811). The dynamics of the skin's immune system. Int. J. Mol. Sci..

[47] Hirabayashi T (2017). PNPLA1 has a crucial role in skin barrier function by directing acylceramide biosynthesis. Nat. Commun..

[48] Williams IR, Kupper TS (1996). Immunity at the surface: Homeostatic mechanisms of the skin immune system. Life Sci..

[49] Fernando IP (2017). Inhibition of inflammatory responses elicited by urban fine dust particles in keratinocytes and macrophages by diphlorethohydroxy-carmalol isolated from a brown alga ishige okamurae. Algae.

[50] Mariathasan S, Monack DM (2007). Inflammasome adaptors and sensors: Intracellular regulators of infection and inflammation. Nat. Rev. Immunol..

[51] Lopez-Castejon G, Brough D (2011). Understanding the mechanism of IL-1β secretion. Cytokine Growth Factor Rev..

[52] Tanaka T, Narazaki M, Kishimoto T (2014). Il-6 in inflammation, immunity, and disease. Cold Spring Harb. Perspect. Biol..

[53] Junek R (2009). Bimodal effect of humic acids on the LPS-induced TNF-α release from differentiated U937 cells. Phytomedicine.

[54] Chen D-H, Liu J-J, Lu F-J, Yang M-L, Yasnang L, Huang T-S (2002). The effect of humic acid on the adhesibility of neutrophils. Thromb. Res..

[55] Liao W, Lin JX, Leonard WJ (2013). Interleukin-2 at the crossroads of effector responses, tolerance, and immunotherapy. Immunity.

[56] Cozzolino V, Monda H, Savy D, Di Meo V, Vinci G, Smalla C (2021). Cooperation among phosphate-solubilizing bacteria, humic acids and arbuscular mycorrhizal fungi induces soil microbiome shifts and enhances plant nutrient uptake. Chem. Biol. Technol. Agric..

[57] Canellas LP, Olivares FL (2017). Production of border cells and colonization of maizeroot tips by *Herbaspirillum seropedicae* are modulated by humic acid. Plant Soil.

[58] Lembo S, Di Caprio R, Balato A, Bigioni AR, Caiazzo G, Schiattarella M, Monfrecola G (2017). Azeloglycine and nicotinamide down-regulate pro-inflammatory mediators in vitro. G Ital. Dermatol. Venereol..

[59] Tanaka Y, Uchi H, Hashimoto-Hachiya A, Furue M (2018). Tryptophan photoproduct FICZ Upregulates IL1A, IL1B, and IL6 expression via oxidative stress in keratinocytes. Oxid. Med. Cell Longev..

[60] Vitiello, G., Venezia, V., Verrillo, M., Nuzzo, A., Houston, J., Cimino, S., D'Errico, G., Aronne, A., Paduano, L., Piccolo, A., Luciani, G. Hybrid humic acid/titanium dioxide nanomaterials as highly effective antimicrobial agents against gram(-) pathogens and antibiotic contaminants in wastewater. *Environ Res.* **193**, 110562. 10.1016/j.envres.2020.110562 (2021). 10.1016/j.envres.2020.110562 33271143

