# Supplementary material for: Antiflammatory activity and potential dermatological applications of characterized humic acids from a lignite and a green compost
Source: Sci Rep. 2022 Feb 9;12:2152. doi: 10.1038/s41598-022-06251-2 (PMC8828863; doi:10.1038/s41598-022-06251-2)
Supplement: Supplementary file 1 — Supplementary Information. [file 41598_2022_6251_MOESM1_ESM.docx]

**Antiflammatory activity and potential dermatological applications of characterized humic acids from a lignite and a green compost**

Mariavittoria Verrillo^1*#^, Melania Parisi^2#^, Davide Savy^1^, Giuseppina Caiazzo^2^, Roberta Di Caprio^2^, Maria Antonietta Luciano^3^, Sara Cacciapuoti^2^, Gabriella Fabbrocini ^2Ɏ^, Alessandro Piccolo^1*Ɏ^

1. Centro Interdipartimentale di Ricerca per la Risonanza Magnetica Nucleare per l’Ambiente, l’Agroalimentare, ed i Nuovi Materiali (CERMANU), Università di Napoli Federico II, Via Università 100, 80055 Portici, Italy
2. Department of Clinical Medicine and Surgery, Università di Napoli Federico II
3. Department of Public Health, Università di Napoli Federico II

Corresponding Authors*: [mariavittoria.verrillo@unina.it](mailto:mariavittoria.verrillo@unina.it); [alessandro.piccolo@unina.it](mailto:alessandro.piccolo@unina.it)

Centro Interdipartimentale di Ricerca per la Risonanza Magnetica Nucleare per l’Ambiente, l’Agroalimentare, ed i Nuovi Materiali (CERMANU), Università di Napoli Federico II,

Via Università 100, 80055 Portici, Italy

# These authors contributed equally to this work

Ɏ These authors contributed equally to this work

**SUPPORTING MATERIALS**

**Table S1**

List of the products released by **HA-LIG** thermochemolysis.

| RT^a^ | Assignment^b^ | | RT | Assignment |
| --- | --- | --- | --- | --- |
| 5.7 | Benzene, 1-CH3O 4(C) | | 20.4 | Naphthalene -(C)3 |
| 5.9 | Benzene-(C)3 | | 21.1 | Fluorene |
| 6.4 | Benzene-(C)3 | | 21.5 | Alkene/Alkane |
| 7.9 | Benzene, 1-(C2) 4-CH3O | | 21.6 | Naphthalene, -(C)3 |
| 8.4 | C8 FAME | | 22.2 | Indole (C)4 |
| 9.3 | Indene, 3-Methyl |  | 23.0 | 3(C)-Hexadecane |
| 9.4 | di CH3O Benzene | | 23.2 | 4(C)-Hexadecane |
| 9.6 | di CH3O Benzene | | 23.4 | Naphthalene, -(C)4 |
| 10.1 | Naphthalene | | 23.8 | Naphthalene, -(C)4 |
| 10.2 | Methyl-Undecane | | 24.2 | Alkene/Alkane |
| 10.6 | N Compound | | 24.9 | C14 FAME |
| 12.4 | Alkyl (C5) Benzene | | 25.3 | Anthracen |
| 12.9 | Methyl Dodecane | | 26.4 | Phenanthren |
| 13.0 | Indene, 1-Ethylidene | | 26.5 | Alkene/Alkane |
| 13.4 | Naphthalene, 1(C) | | 28.9 | (C)-Octadecane |
| 13.6 | C10 FAME. | | 29.4 | Nonadecane |
| 14.9 | Indole, 1,4-(C)2 | | 30.0 | C16 FAME |
| 15.2 | Alkyl (C6) Benzene | | 30.7 | Phenanthrene 3(C) |
| 15.7 | N Compound | | 31.8 | Eicosane |
| 15.8 | Tetradecane | | 34.1 | Heneicosane |
| 16.1 | Naphthalene, -(C)2 | | 34.7 | C18 FAME |
| 16.5 | Naphthalene, -(C)2 | | 36.3 | Docosane |
| 16.6 | Pyrido[D]Pyrimidine 4-CH3O | | 38.4 | Alkene/Alkane |
| 17.3 | Alkyl (C7) Benzene | | 40.5 | Alkene/Alkane |
| 17.5 | Naphthalene, -(C)2 | | 42.4 | Alkyl Pyrene |
| 17.6 | Methyl Tetradecane | | 44.3 | Alkene/Alkane |
| 17.9 | Methyl Tetradecane | | 46.2 | Alkene/Alkane |
| 18.3 | Naphthalen, (C), (C2) | | 47.9 | Alkene/Alkane |
| 18.7 | Alkene/Alkane | | 49.6 | Alkene/Alkane |
| 19.3 | Dibenzofuran | | 51.3 | Alkene/Alkane |
| 19.5 | Indole, 1,2,3-(C)3 | | 53.1 | Alkene/Alkane |
| 19.6 | Naphthalene, -(C)3 | | 55.9 | Alkene/Alkane |
| 20.2 | Naphthalene, -(C)3 | | 58.0 | Alkene/Alkane |
|  |  |  |  |  |

a. RT = Retention Time (minutes)

b. (C)=methyl; (C_2_)=ethyl; (C_3_)=propyl; FAME= fatty acid methyl ester

**Table S2**

List of the products released by **HA-CYN** thermochemolysis.

| RT^a^ | Assignment | RT | Assignment |
| --- | --- | --- | --- |
| 8.82 | Phenol. 2-Ethyl- | 24.52 | Methyl 13-Methyltetradecanoate |
| 9.71 | 2.3-Dimethoxytoluene | 24.74 | Benzenesulfonamide. N-Butyl- |
| 10.48 | 1h-Indole. 1-Me | 25.97 | Caffeine |
| 10.7 | Phenol. 4-Ethyl-2-Methoxy- | 26.81 | 2-Propenoic Acid. 3-3.4-Dimethoxyphenyl |
| 11.41 | Indole | 27.04 | Tetradecanoic Acid. 10.13-Dimethyl-. Me |
| 11.73 | 2-Methoxy-4-Vinylphenol | 27.37 | Methyl Hexadec-9-Enoate |
| 12.86 | Phenol. 2.6-Dimethoxy- | 27.62 | Ethyl 9-Hexadecenoate |
| 13.43 | Benzoic Acid. 3-Methoxy-. Me | 28.01 | Hexadecanoic Acid. Me |
| 13.75 | Indolizine. 7-Methyl- | 28.97 | Methyl 10-Methyl-Hexadecanoate |
| 13.97 | Benzene. 1.2.3-Trimethoxy-5-Methyl- | 29.46 | Methyl 15-Methylhexadecanoate |
| 14.15 | Phenol. 2-Methoxy-4-(1-Propenyl)-. (Z)- | 29.65 | Heptadecanoic Acid. Me |
| 14.57 | 1h-Isoindole-1.3(2h)-Dione. 2-Methyl- | 29.91 | Methyl 14-Methylhexadecanoate |
| 14.98 | 1h-1-Benzazepine. 2.3.4.5-Tetrahydro- | 30.33 | Methyl 15-Methylhexadecanoate |
| 15.29 | 1.2.4-Trimethoxybenzene | 31.81 | Methyl 12.15-Octadecadienoate |
| 15.41 | Phenol. 2-Methoxy-4-(1-Propenyl)-. (Z)- | 31.99 | Methyl 11-Octadecenoate |
| 15.63 | Benzoic Acid. 3-Hydroxy-. Me | 32.12 | Methyl 11-Octadecenoate |
| 16.28 | Benzonitrile. 2.4.6-Trimethyl- | 32.64 | Octadecanoic Acid. Me |
| 16.64 | 2.5-Dimethoxyethylbenzene | 34.48 | Methyl 9.10-Methylene-Octadecanoate |
| 16.9 | 1h-Indole-2.3-Dione. 1-Methyl-. 3-Hydraz | 38.21 | Hexanedioic Acid. Bis(2-Ethylhexyl) Este |
| 17.41 | Benzoic Acid. 4-Hydroxy-3-Methoxy-. Me | 40.94 | Methyl 20-Methyl-Heneicosanoate |
| 17.44 | Dodecanoic Acid. Me | 42.33 | Pentacosane |
| 18.03 | 1h-Indole-2.3-Dione. 1-Methyl-. 3-Hydrazine | 42.83 | Methyl 20-Methyl-Docosanoate |
| 18.64 | Ethanone. 1-(3.4-Dimethoxyphenyl)- | 43.39 | Docosanoic Acid. 2-Hydroxy-. Me |
| 18.9 | Benzoic Acid. 4-Hydroxy-3-Methoxy-. Meth | 44.16 | Hexacosane |
| 19.35 | Benzoic Acid. 3.4-Dimethoxy-. Me | 44.68 | Tetracosanoic Acid. Me |
| 19.61 | Benzene. 1.1'-Propylidenebis- | 46.43 | Methyl 17-Methyl-Tetracosanoate |
| 20.78 | Benzene. 1.2.3-Trimethoxy-5-(2-Propenyl) | 47 | Methyl 2-Hydroxy-Tetracosanoate |
| 21.64 | Ethanone. 1-(3.4.5-Trimethoxyphenyl)- | 48.16 | Hexacosanoic Acid. Me |
| 22.58 | Phenol. 2.6-Dimethoxy-4-(2-Propenyl)- | 49.28 | Stigmasta-5.22-Dien-3-Ol. Acetate. (3.Be |
| 22.76 | Benzoic Acid. 3.4.5-Trimethoxy-. Methyl | 50.25 | Stigmastan-3.5-Diene |
| 22.85 | Tridecanoic Acid. 12-Methyl-. Me | 50.92 | Dotriacontane |
| 24.15 | Benzoic Acid. 4-Hydroxy-3.5-Dimethoxy | 51.42 | Methyl Octacosanoate |
|  |  | 54.51 | Methyl Triacontanoate |

a. RT = Retention Time (minutes)

**
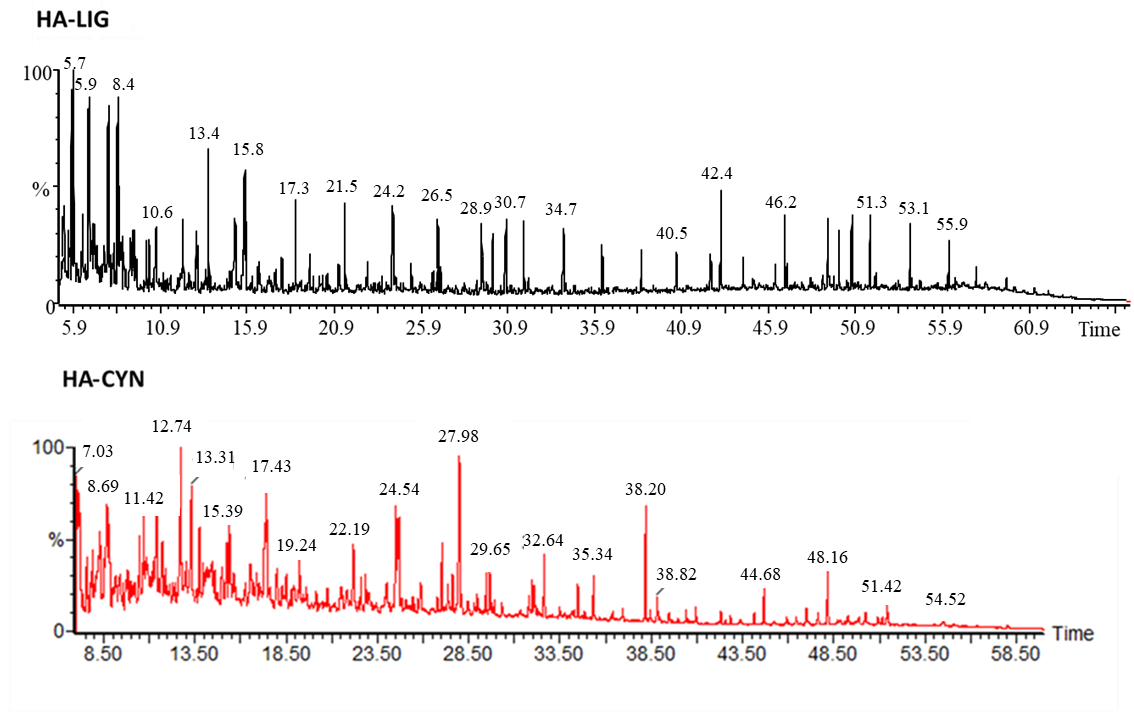
**

**Figure S1:** Pyr-GC-MS chromatogram of HA-LIG (black) and HA-CYN (red) samples.

**
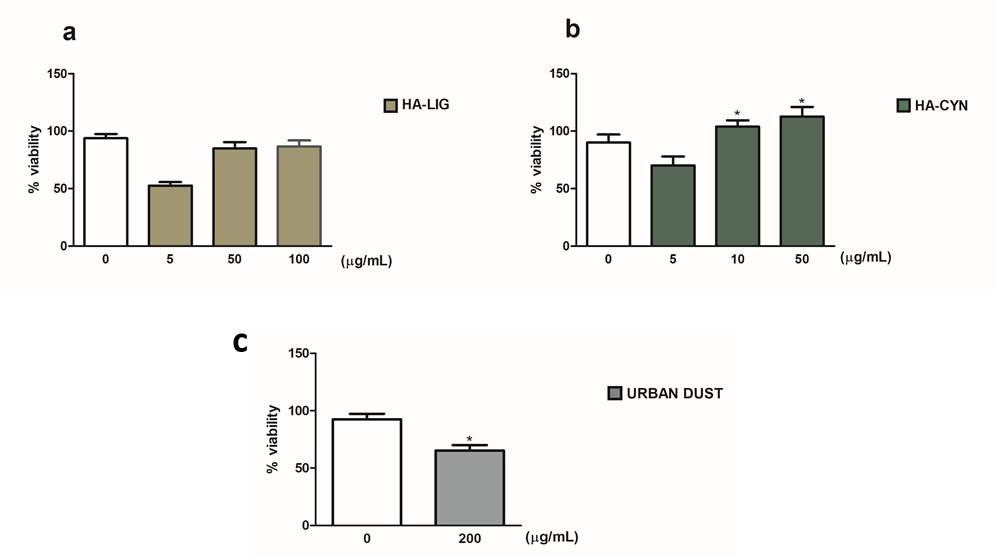
**

**Figure S2**: Cell viability assessment by Trypan blue coloration 4 h after the addition of (a) different concentrations of and HA-LIG (5, 50 and 100 µg.mL^-1^), (b) HA-CYN (5, 10 and 50 µg.mL^-1^) and (c) Urban Dust (0 and 200 µg.mL^-1^). Cellular viability rate and statistical significance were determined with respect to 100% viability of untreated control cells. Data are expressed as mean (± SD) of three independent experiments, each performed in triplicate. Student’s t-test was used to calculate significant differences (*P < 0.05).
